# Association between asthma and polyunsaturated fatty acids intake in Brazilian adolescents: study of cardiovascular risk in adolescents-ERICA

**DOI:** 10.3389/fpubh.2025.1549750

**Published:** 2025-07-04

**Authors:** Érica Azevedo de Oliveira Costa Jordão, Mara Morelo Rocha Felix, Marcia Takey, Denise Tavares Giannini, Maria Cristina Caetano Kuschnir, Fábio Chigres Kuschnir

**Affiliations:** Universidade do Estado do Rio de Janeiro (UERJ), Programa de Pos-graduação em Ciências Médicas (PGCM), Rio de Janeiro, State of Rio de Janeiro, Brazil

**Keywords:** adolescent, asthma, cross-sectional studies, fatty acids omega-6, fatty acids omega-3, fatty acids unsaturated, eicosapentaenoic acids, docosahexaenoic acids

## Abstract

**Introduction:**

Previous studies have shown that asthma is associated with a less traditional diet pattern with an unbalanced polyunsaturated fatty acids (PUFAs) distribution. This study aimed to investigate the association between asthma and PUFA intake in Brazilian adolescents.

**Methods:**

This is a cross-sectional study, using data from the Study of Cardiovascular Risks in Adolescents-ERICA, a national, school-based multicenter survey with a sample representative of Brazilian adolescents (12–17 years old). The presence of at least one wheezing attack in the last 12 months defined asthma. The intake of the following PUFAs was evaluated: alpha-linolenic acid (ALA), eicosapentaenoic acid (EPA), docosahexaenoic acid (DHA), linoleic acid (LA) and arachidonic acid (ARA) as well as N6N3 ratio (the sum of LA and ARA over ALA, DHA and EPA). The odds ratio (OR) and the respective 95% Confidence Interval (95% CI) between asthma and PUFAs intake, as well as other study variables was calculated using Logistic Regression.

**Results:**

A total of 64,904 participants were included for the analysis. After adjustment, asthma remained significantly associated with ALA (OR:1.05; CI95%:1.02–1.09) and EPA (OR:0.61; 95%CI:0.39–0.95), being the first a positive association and the latter a negative one. There were no significant association between asthma and N6N3 ratio, as well as the other PUFAs evaluated.

**Conclusion:**

Our findings reveal a higher chance of asthma among Brazilian adolescents with increased ALA consumption and, elevated intake of EPA was associated with decreased odds.

## Introduction

Asthma is the most common chronic non-communicable disease among children and adolescents (1, 2). Most consensus on asthma highlight adolescence as a critical period in which the clinical and epidemiological characteristics of the disease go through significant change (3), giving the opportunity for interventions that may improve outcomes well into adulthood.

In the last few decades asthma prevalence has increased, following changes in lifestyle (4–6), including diet patterns that have undergone a shift, becoming richer in ultra-processed foods and poorer in fish, fresh fruits and vegetables (7–9). These changes have led to a higher intake of saturated and trans fatty acids, plus an unbalanced essential polyunsaturated fatty acids (PUFAs) distribution, particularly among the younger population (10, 11).

Fatty acids are important part of many lipidic molecules and a major component of dietary fats, with an important role as substrate for the production of inflammatory mediators (12–15). They are characterized by a carboxylic acid with an aliphatic tail (carbon chain) (13). The presence of double bonds on the carbon chain defines the fatty acid as saturated (no double bonds), monounsaturated (one double bond) or polyunsaturated (more than one double bond) (13, 15, 16). The polyunsaturated fatty acids (PUFAs) are classified within the omega 6 (N6) or omega 3 (N3) series according to the location of the first double bond in the carbon chain starting from the methyl end, being the linoleic acid (LA) and alpha-linolenic acid (ALA) the simpler components of each series (13, 15–17). Arachidonic acid (ARA), from the N6 series, is a substrate to yield eicosanoid family mediators, such as leukotrienes and prostaglandins with pro-inflammatory activity (14, 18–20). Conversely, eicosapentaenoic acid (EPA) and docosahexaenoic acid (DHA), from the N3 series, are precursors of pro-resolving lipid mediators with an anti-inflammatory profile (12, 14, 18–21).

Dietary intake of PUFAs influences the levels of these fatty acids in the phospholipids of cell membranes in macrophages, neutrophils, and lymphocytes (22–24). Thus, a higher N3 intake would lead to an increased presence of these fatty acids in cell membranes and, consequently, greater availability for the production of pro-resolving lipid mediator, thereby steering the inflammatory response toward resolution. Therefore, the balance in the N3 and N6 fatty acids intake may play a role in modulating inflammatory response, with N6 promoting a pro-inflammatory pattern while N3 performs an opposite function in this balance (12, 14, 16, 19–21). Considering that asthma is almost always characterized by inflammation (2), it has been speculated that the N3 and N6 intake may be associated with the development or worsening of asthma control (16).

Traditional diets, generally richer in vegetables, fresh fruits, fish and seafood, and thus, with larger portions of protein, fiber, antioxidants and unsaturated fats, seems to be associated with a lower prevalence of asthma (25–32), whilst diets richer in processed foods, hence, high in saturated and trans fatty acids, with a higher prevalence (33–36). Meanwhile, studies evaluating the consumption of PUFAs in asthma still have inconsistent results (16, 26, 37–43).

Therefore, the objective of the present study was to investigate the association between the consumption of PUFAs, N3 (ALA, EPA and DHA), N6 (LA and ARA) and the N6/N3 ratio in the diet and asthma in Brazilian adolescents.

## Methods

### Study design and population

This is a cross-sectional study, using the database of the Study of Cardiovascular Risks in Adolescents (Portuguese acronym- ERICA), a national, school-based multicenter survey, which primary goal was to evaluate the prevalence of metabolic syndrome and cardiovascular risk factors among Brazilian adolescents. Data from approximately 74,000 students (12–17 years) living in municipalities with more than 100 thousand inhabitants were evaluated, excluding pregnant women or those with disabilities that would hinder anthropometric measurements. The ERICA sample design has already been described in previous publications (44, 45).

### Data collection

Were included for the analysis those participants who completed: (1) the self-completed questionnaire that comprised sociodemographic data, and questions about, physical activity, and the presence of chronic diseases, such as asthma, among other issues (46); (2) the 24-h dietary recall, applied by trained researchers using ERICA-Rec24h, a software specifically designed for the ERICA survey, to register food consumption (10, 46, 47) and (3) the anthropometric measurements. Adolescents who did not answer the question of interest for the definition of asthma or did not complete the dietary recall were excluded.

Asthma was defined by the question: “In the last 12 months, how many wheezing attacks (wheezing) did you have?,” from the validated Brazilian version of the ISAAC questionnaire. Those participants who reported at least one wheezing attack in the last 12 months, were considered as having asthma (48, 49). This question has been shown to be the best tool to discriminate asthma in epidemiologic studies (50).

The ERICA-rec24h was developed using the database from the Brasil-Nutri software, created to standardize the input of food intake data in Brazil (10, 46, 47). The nutrients and energy intake data were estimated based on the Brazilian Food Composition Table (51) and Brazilian Portion Size Table (52). The use of supplements or medications were not included for the nutrient intake data (10, 47).

The PUFAs, AL, ALA, EPA, DHA, and ARA intakes were estimated in grams per day (g/day), as continuous variables as well as total and saturated fat intake. The energy intake was estimated in kilocalories (kcal) per day. Finally, the N6/N3 ratio was obtained by dividing the sum of LA plus ARA over the sum of ALA plus DHA and EPA.

The sociodemographic variables collected were: sex (feminine/masculine), skin color (white or non-white) and age that was categorized in two groups (12–14 years/15–17 years). School administration (public or private) and location (urban or rural) was also collected (46).

Adolescents with less than 300 min of physical activity per week were classified as sedentary, and those with 300 min or more were active (53).

Anthropometric measurements were collected by trained researchers according to written standardized procedures with the adolescent barefoot and wearing light clothing. Weight was measured on a portable digital scale (Líder^®^, São Paulo, Brazil), and height using a portable stadiometer with variation of 1 mm, (Alturexata^®^, Minas Gerais, Brazil), the mean of the two measurements of weight and height were used for the analysis (46, 54). The nutritional status was defined based on the Body mass index (BMI)-for-age Z-score curves. Those with a Z-score below −2 were classified as very low/low weight, greater than −2 and lower than +1 are eutrophic, and, overweight/obese if z-score is equal +1 or more (55, 56).

Waist circumference (WC) was measured horizontally, with the individual at the upright position, using a fiberglass tape, with 1.5 m length and millimeter resolution (Sanny^®^, São Paulo, Brazil), at half the distance between iliac crest and lower costal margin (46, 54). For the definition WC adequacy, the cutoff points based on the International Diabetes Federation (IDF) reference table (57) were used. Since the IDF’s 90th percentile for girls aged 12–17 years were higher than the cutoff points for adults, the later were used (80 cm). For boys, the cut points were as follows: (1) 12 years: 84.8 cm; (2) 13 years: 88.2 cm; (3) 14–17 years: 90.0 cm, the same as adults, since the 90th percentile for this age group is higher.

### Statistical analysis

Statistical analyses were performed using the Stata program, version 18.0 (StataCorp LP, College Station, TX, United States), using the set of commands for analyzing survey data in a complex sample. Data analysis was descriptive and inferential, using statistical tests that allow analyzing possible associations between the evaluated outcomes (asthma) and the exposure under study (consumption of PUFAs, ALA, DHA, EPA, LA and ARA and N6/N3 ratio).

The prevalence of asthma and its respective 95% confidence intervals (95%CI) was calculated according to sex, age group, skin color, type and location of school (public or private), nutritional status and physical activity. In addition, the mean consumption of PUFAs (ALA, LA, EPA, DHA, ARA, and N6N3 ratio), energy, total and saturated fat along with the 95%CI was estimated according to asthma status.

For the bivariate analysis between asthma and PUFAs intake, as well as the other study variables (sex, age group, type of school, nutritional status, physical activity, total and saturated fat intake and energy), the odds ratio (Odds Ratio—OR) and the respective 95%CI was estimated using Logistic Regression. A one-way ANOVA was performed to compare the effect of PUFAs intake on the other study variables.

Those that were associated with both the outcome and exposure with *p* < 0.20 were considered confounding factors and included in the multivariate models. Associations with a *p*-value <0.05 were considered significant.

Additionally, for the PUFAs that showed association with asthma in the multivariate model, we used the inverse-probability-weighted regression adjustment to estimate the treatment effects, allowing to control the cofounding variables for both the outcome (asthma) and exposure.

### Ethical aspects

ERICA was carried out in accordance with the principles of the Helsinki Declaration. The project was approved by the Research Ethics Committee of the Institute of Collective Health Study *Instituto de Estudos de Saúde Coletiva da Universidade Federal do Rio de Janeiro* (IESC/UFRJ) (process number 45/2008) and subsequently approved in each participating center. Student privacy and data confidentiality were guaranteed throughout the study. Each participant signed the assent form and, additionally, the informed consent was collected from their legal guardians when required by the local Research Ethics Committee.

## Results

**Figure 1:**
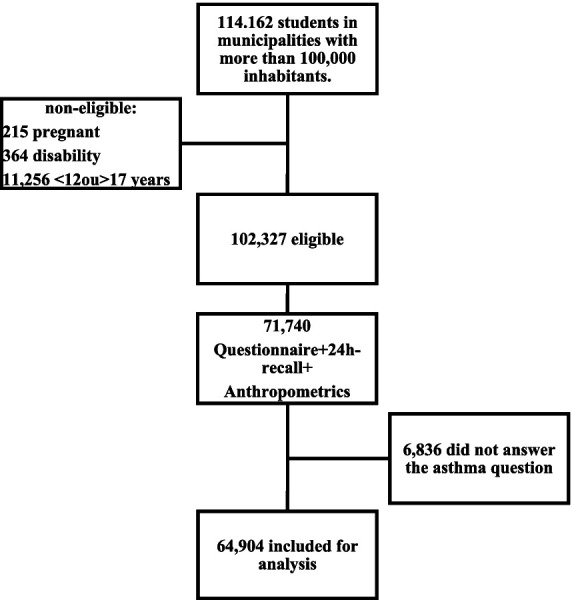
Flow chart showing sample selection. Adapted from Da Silva et al. (45).

A total of 64,904 individuals were included for the analysis (Figure 1). The prevalence of asthma was 14.5%, 49.5% were female and 47.3% were 15–17 years old. Overweight/obese students made up a total of 25.4% of the sample. Table 1 shows the general characteristics of the sample.

**Table 1:** Overall characteristics of the participants according to presence of asthma.

*p*-value	Total	Asthma	Nonasthma	*p*-value
	%(95%CI)	% (95%CI)	%(95%CI)	
		14.5 (13.5–15.5)	85.5 (84.5–86.5)	
15–17 years	47.3 (47.3–47.3)	14.8 (13.7–16.1)	85.2 (83.9–86.3)	0.000*
Feminine	49.8 (49.8–49.8)	16.6 (15.4–17.9)	83.4 (82.1–84.6)	0.000*
White	40.2 (38.5–41.8)	16.5 (14.5–18.7)	83.5 (81.3–85.5)	0.000*
Public school	17.5 (13.6–22.2)	17.6 (15.8–19.7)	82.4 (80.3–84.3)	0.000*
Urban school	96.1 (88.1–98.8)	14.1 (13.4–15)	85.9 (85.0–86.7)	0.000*
Overweight/obese	25.4 (24.4–26.5)	15.7 (14.3–17.2)	84.3 (82.8–85.7)	0.000*
Elevated WC	10.9 (10.2–11.6)	17.1 (15.1–19.3)	82.9 (80.7–84.9)	0.000*
Sedentary	48.0 (47–49.0)	13.8 (12.4–15.3)	86.2 (84.7–87.6)	0.021*

	Mean (95%CI)	Mean (95%CI)	Mean (95%CI)	
Energy (k/day)	2,288 (2,244–2331)	2,341 (2,238–2,443)	2,279 (2,240–2,318)	0.099**
Total fat (g/day)	77.91(76.26–79.56)	80.38 (76.47–84.28)	77.49 (75.99–78.99)	0.006**
Saturated fat (g/day)	28.25 (27.71–28.8)	29.29 (28.09–30.48)	28.08 (27.53–28.62)	0.000**
ALA intake (g/day)	1.7 (1.65–1.75)	1.77 (1.62–1.93)	1.68 (1.65–1.72)	0.003**
DHA intake (g/day)	0.07 (0.07–0.08)	0.08 (0.06–0.10)	0.07 (0.06–0.08)	0.673**
EPA intake (g/day)	0.02 (0.02–0.02)	0.02 (0.02–0.03)	0.02 (0.02–0.02)	0.026**
LA intake (g/day)	13.45 (13.02–13.87)	14.04 (12.78–15.3)	13.35 (13.02–13.67)	0.036**
ARA intake (g/day)	0.15 (0.15–0.16)	0.16 (0.14–0.17)	0.15 (0.15–0.16)	0.054**
N6N3	7.8 (7.74–7.86)	7.76 (7.66–7.85)	7.81 (7.75–7.87)	0.118**

*Logistic regression. **Anova; ALA, alfa-linolenic acid intake (grams/day); DHA, docosahexaenoic acid intake (grams/day); EPA, eicosapentaenoic acid intake (grams/day); LA, linoleic acid intake (grams/day); ARA, arachidonic acid intake (grams/day); N6N3, ratio of (LA plus ARA) to (ALA plus DHA and EPA).

The mean intake of the PUFAs and N6N3 ratio, energy, total and saturated fat according to asthmais shown in Table 1.

In the bivariate analysis, asthma was significantly associated with ALA (OR: 1.03; 95%CI: 1.01–1.05) and EPA (OR: 0.63; 95%CI: 0.42–0.94), being the first a positive association and the latter a negative one. There were no significant association with the other PUFAs or with N6N3 ratio. The one-way ANOVA revealed that the mean value of ALA was associated with a *p* < 0.2 with all possible cofounders except for school administration (*F*-value: 0.02; p: 0.9). Similarly, EPA was associated with all study variables except for nutritional status (*F*-value: 1.63; p: 0.2). Therefore, these variables were excluded from the multivariable model for each PUFA.

**Figure 2:**
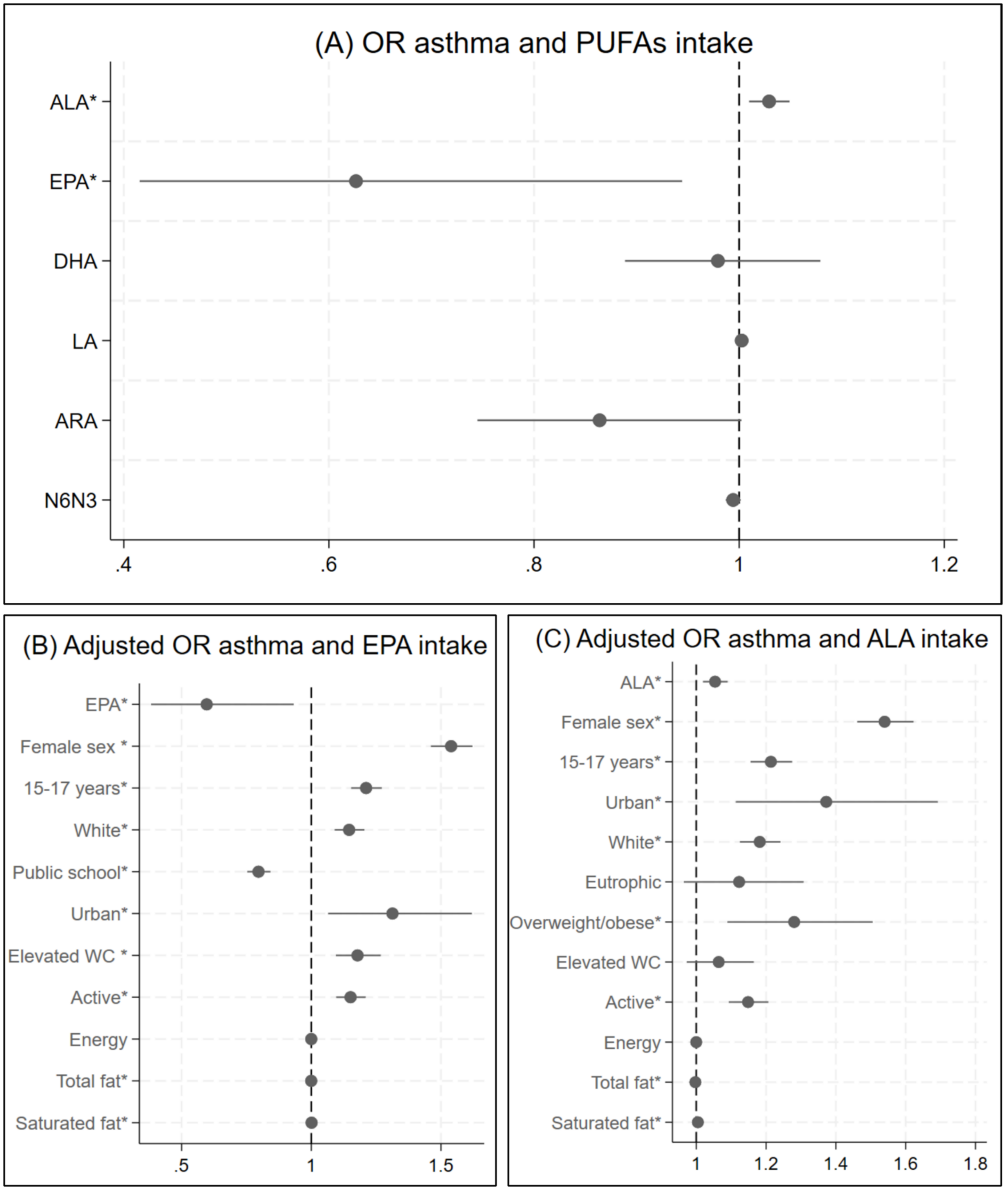
Logistic regression asthma and PUFAs intake. **(A)** Odds ratio and corresponding 95% confidence interval between asthma and PUFAs intake (bivariate analysis). **(B)** Odds ratio and corresponding 95% confidence interval between asthma and EPA intake adjusted for age group, sex, skin color, school administration and location, waist circumference, physical activity, total fat and saturated fat. **(C)** Odds ratio and corresponding 95% confidence interval between asthma and ALA intake adjusted for age group, sex, skin color, school location, waist circumference, physical activity, total fat and saturated fat. *p < 0.05.

For the multivariate models, age group, sex, skin color, school location, nutritional status, waist circumference, physical activity, energy, total fat and saturated fat were included for ALA (OR: 1.05; 95%CI: 1.02–1.09), and age group, sex, skin color, school administration and location, waist circumference, physical activity, energy, total and saturated fat for EPA (OR: 0.61; 95%CI: 0.39–0.95), neither association lost its significance (Figure 2).

In the sensitivity analysis, the students with asthma had a mean ALA intake of 1.67grams/day (g/day) (95%CI: 1.66–1.69) and those with no asthma a 1.64 g/day (95%CI: 1.64–1.66), *p* < 0.001. The mean EPA consumption was 0.26 g/day (95%IC: 0.26–0.27) in the nonasthma group and 0.24 g/day (95%IC: 0.23–0.25) in the asthma group (*p* < 0.001).

## Discussion

Asthma was associated with a higher intake of ALA, whereas the odds of asthma were lower in the group with higher consumption of EPA in our study.

In this sample, the prevalence of asthma was 14.5%, slightly higher than the 13.1% reported in the Study of Cardiovascular Risks in Adolescents (ERICA) (48, 49), after excluding participants who did not complete the 24-h food recall. Previous studies have shown increase in asthma prevalence in the last few decades, followed by stabilization in recent years (4–6). These findings may be attributed to lifestyle changes, including eating habits, that occurred in recent decades due to globalization and urbanization.

Following the pattern of developing countries, Brazilians have increased the total calories from ultra-processed foods among foods purchased for consumption between the years of 1987 to 2012, rising from 18.7 to 26.1% (9). Additionally, there has been a decreased intake of favorable fats with a higher than recommended intake of saturated and trans fatty acids leading to an imbalance in the N6N3 ratio (11). Data from ERICA showed a high prevalence of ultra-processed foods consumption and a low prevalence of fruits among Brazilian adolescents. Notably, fish did not appear among the 20 most consumed foods in any age group, and in no Brazilian macro-region (10). The impact of fish (26) and fruits and vegetables intake (27, 31) in asthma has been previously demonstrated, in meta-analysis.

A healthy diet pattern, evaluated through different scores and definitions, seems to be protective for asthma (32, 58–60). A prospective study evaluating Puerto Rican children for 1 year found increased odds of asthma among those with an unhealthy diet, although no change in lung function was observed (59). Interestingly, a study following participants since birth found a better lung function in individuals with a health-conscious diet in mid-childhood (32). The different results may be attributed to the smaller sample size and shorter follow-up period in the former study.

In a recent study from the National Health and Nutrition Examination Survey NHANES, which evaluated dietary patterns and respiratory health in adults, total protein was the food group that most influenced the asthma risk, possibly because almost two thirds of the study population was older than 40 years old and protein is an important indicator of overall nutrition status (30). Diet Inflammatory Index (DII) which predicts the inflammatory potential of the diet and has N3 intake as one of its components, showed no association with asthma (30). However, a study evaluating only the DII and asthma also using data from NHANES, but in a slightly younger population found a significant association (61). Indicating that the food components that influence asthma risk may vary accordingly with age.

The mean consumption of EPA and DHA in our sample was similar to other studies. A systematic review carried out in 2011, by Harika et al., evaluating different countries (Brazil was not included), found EPA intake of was 0.01 to 0.06 g/day and of DHA 0.03–0.12 g /day (62). Additionally, the mean N6N3 ratio was above the minimal recommended values of 4:1–5:1 (11).

Contrary to our initial hypothesis that an increased ALA intake would be protective for asthma, especially in contrast to the LA intake translating into a lower N6/N3 ratio, we observed a greater chance for asthma in the higher ALA intake group and no association between asthma and N6N3 ratio. However, there was a lower chance of having asthma in the group with higher EPA intake.

Previous studies have investigated the relation on N6 intake and asthma with conflicting results. Miyake et al. observed a higher chance for asthma and wheeze in the highest N3 and N6 consumption groups in Japanese children aged 6 to 15 years old, this association lost its significance for asthma in the multivariate model, remaining only for LA and N6 consumption (40). In a prospective study in Sweden, asthma with 24 years old was associated with higher intake of LA at 8 years (OR: 1.41; 95%CI: 1.10–1.82) and ARA at 16 years (OR: 1.32; 95%CI: 1.02–1.70) (42). More recently, a cross-sectional study using data from NHANES, involving children and adolescents (6–19 years) found a negative association between asthma and N6 intake up to a threshold beyond which no further decrease in the asthma risk was observed (41). In contrast, a previous study in children aged 5 to 12 years old found a positive association between N6 consumption, and asthma severity, as well as an enhanced response to indoor pollution among asthmatic patients, whereas N3 intake was associated with a reduced effect of indoor pollution on asthma (43). Suggesting that diet and environmental exposures may interact modulating asthma severity.

Conversely, asthma was inversely associated with a higher N3 intake in a prospective study carried out in young adults aged between 18 and 30 years old from the Coronary Artery Risk Development in Young Adults (CARDIA) study (39). Similar results were observed in a study evaluating 14,727 children aged 2–12 years from the NHANES which reported a lower chance of asthma in individuals with higher intake of DHA, EPA and total N3, but only after adjusting for energy, total fat, maternal smoking during pregnancy, birth weight, nutritional status, age, skin color, sex and family poverty income ratio (38).

In the same year another study involving participants from the NHANES, evaluated 8.835 children and adolescents up to 20 years of age, and found an inverse correlation between N3 intake and the prevalence of asthma, however, only up to 59 mg/kg/day (OR = 0.984, 95%CI: 0.977–0.991, *p* < 0.001), from this point on, there was no significant association (37).

ALA and LA are essential fatty acids found in vegetable oils and seeds (17), being the precursor PUFA in their respective series, they are catalyzed by elongases and desaturases into longer chain PUFAs (EPA, DHA, ARA, among others) (12, 14–17). The N6 and N3 PUFAs compete for the same enzymes in this process and higher EPA or DHA intake appears to decrease the conversion of ALA which does not increase with higher ALA intake (15, 63). Besides, dietary LA does not seem to change tissue ARA in adults (64). Therefore, the availability of PUFAs in plasma and tissues does not necessarily mirror their ALA and LA dietary intake.

The conversion of ALA and LA dependents on the efficiency of enzymes (15, 65, 66) encoded by genes like FADS1 and FADS2 with polymorphisms linked to higher ALA levels and lower EPA and DHA levels, while variations in ELOVL2 affects EPA, DPA and DHA levels in populations of European ancestry (66).

Talaei et al., in a longitudinal study, observed an inverse association between the consumption of EPA and DHA and the incidence of asthma only in the group of children with a common genetic variant of FADS associated with a lower efficiency in the conversion of ALA into longer-chain fatty acids (65). Those studies were carried out in populations with European ancestry, and so we cannot infer that those polymorphisms have such influence in our sample, once, to our knowledge, there are no studies evaluating the influence of these genetic variables on asthma in our population.

Many factors may contribute to the inconsistency observed within studies in this field. Beyond host individual features, the lack of a standardized method to assess PUFA intake, the wide diversity of dietary surveys used across the studies, and the variety of databases from which the nutritional values of foods are derived also contribute. Moreover, the N6N3 ratio does not necessarily translates the plasma or tissue PUFA concentration (15, 63, 64) and there is no consensus regarding the method to calculate the N6N3 ratio and which PUFA should be included (15).

Our study has some limitations that should be taken into consideration. First, those stemming from the cross-sectional design, which does not allow for the establishment of a causal relationship. Also, the estimates of PUFA intake are derived from a 24-h dietary recall answered by the adolescent which may lead to memory errors. However, the dietary interview was performed by trained field researchers equipped to conduct dietary interviews using the multiple-pass method known to reduce those potential biases (67). Moreover, the use of standardized questionnaires and a nationally representative sample size are notable strengths of our study.

## Conclusion

Our findings reveal a higher prevalence of asthma among Brazilian adolescents with increased ALA consumption. Conversely, elevated intake of EPA was associated with decreased odds of asthma. Remarkably, these associations remained statistically significant even after controlling for other variables in the study.

## Data Availability

The data analyzed in this study is subject to the following licenses/restrictions: Data is available upon request for the Study of Cardiovascular Risk in Brazilian Adolescents (ERICA) publication committee (ericapublica@gmail.com). Requests to access these datasets should be directed to ericapublica@gmail.com.
